# Effect of the ethanolic extract and essential oil of *Ferulago angulata* (Schlecht.) Boiss. on protein, physicochemical, sensory, and microbial characteristics of probiotic yogurt during storage time

**DOI:** 10.1002/fsn3.1984

**Published:** 2020-11-04

**Authors:** Mahshid Keshavarzi, Anousheh Sharifan, Seyed Ali Yasini Ardakani

**Affiliations:** ^1^ Department of Food Science and Technology, Science and Research Branch Islamic Azad University Tehran Iran; ^2^ Department of Food Science and Technology Faculty of Agriculture and Natural Resources Yazd Branch Islamic Azad University Yazd Iran

**Keywords:** *Ferulago angulate*, herbal yogurt, physicochemical characteristics, survival of probiotics, yogurt protein

## Abstract

**Background:**

The use of functional food, such as probiotic products, is important due to their health benefits against various diseases. Phenolic and aromatic compounds originating from medical plants can contribute to the growth of probiotic bacteria.

**Methods:**

The ethanolic extract (0.2% and 0.4%) and essential oil (0.01% and 0.03%) of *Ferulago angulata* (FAEE and FAEO, respectively) were added to probiotic yogurt (*Lactobacillus acidophilus* and *Bifidobacterium bifidum* bacteria) to investigate their effects on the survival of probiotic bacteria during storage time (21 days) and assess its physicochemical, protein, and organoleptic properties.

**Results:**

Upon increasing the concentration of FAEE and FAEO, the value of total phenol content, acidity, viscosity, and water absorption of yogurt treatments increased, and the pH, syneresis, and solubility of treatments showed a decreasing trend (*p* < .05). Also, adding 0.01% FAEO and 0.2% FAEE improved the organoleptic properties of yogurt (*p* < .05) compared to the control treatment. The survivability of the investigated probiotic bacteria demonstrated a decreased trend during storage in all treatments, but at the end of the study, the number of both probiotic bacteria in all treatments was significantly higher than that of the control samples.

**Conclusion:**

Based on the results of protein, physicochemical, microbial, and sensory tests of herbal probiotic yogurts, the addition of 0.03% essential oil is the best way to realize the goals of the research.

## Graphical abstract

In this report, the effect of *Ferulago angulata* plant on probiotic yogurt was investigated to observe its results on physicochemical, sensory, and microbial characteristics, the results of which were consistent with the results of some other articles.

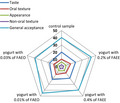

## 1 INTRODUCTION

Probiotics are live microorganisms which, when administered in adequate amounts, confer a health benefit on the host (FAO/WHO, 2001). The popularity of probiotics has continuously grown, and various probiotic food products have been marketed, including probiotic yogurts (Sarvari et al., 2014).

The probiotic yogurt, having probiotic effects, is a product with adjuvant microorganisms. There are numerous advantages associated with consuming fermented dairy products containing probiotic bacteria (Aswal et al., 2011). However, to deliver their health benefits, probiotics must be present in food products above a threshold level (>6 log cfu/g) at the time of consumption in order to survive the passage through the upper and lower parts of the gastrointestinal (GI) tract (Marinaki et al., 2016). Nevertheless, during the storage of probiotic products, the survivability of these bacteria shows a decreasing trend due to several factors, for example, the low pH of fermented foods, hydrogen peroxide produced by some *lactobacilli*, and high oxygen content (Kim et al., 2019; Sarvari et al., 2014). The most commonly used probiotic supplements contain the species of *Lactobacillus* and *Bifidobacterium* and are part of the healthy human intestinal microbiota (Nashaat AL‐Saadi, 2016).

Herbal products (spices, essential oils, and extracts) have been used as a source of functional flavoring agents (Azizkhani & Parsaeimehr, 2018), bioactive antioxidants, and other compounds, such as phenolic compounds, and can be incorporated as nontraditional additives in fermented milk products, including yogurt (Mahmoudi et al., 2016). There are several studies about the health benefits of herbs, including antimicrobial, antioxidant, anti‐inflammatory, and anticarcinogenic properties (Azizkhani & Parsaeimehr, 2018).

Combining probiotics with herbal products may provide further antimicrobial‐therapeutic properties. However, as herbs are antimicrobials, they may affect the viability of probiotic microorganisms. In vitro studies testing herbs on the growth of selected probiotics demonstrated that herbal products significantly enhance the growth of probiotics while inhibiting pathogens (Be et al., 2009; Sutherland et al., 2009). Also, the use of herbal extracts can exert a strong effect on food properties, including structural, functional, and nutritional changes in proteins. Several factors can influence the action and reaction of phenolic compounds, most notably pH, protein type and concentration, and the structure of phenolic compounds. (Ozdal et al., 2013). One way to enhance the viability of probiotic bacteria is the addition of medicinal plants to dairy products which, while increasing the viability of these bacteria and the shelf life of these products, does not adversely affect the organoleptic properties of the products. (Yerlikaya, 2014).

*Ferulago angulata* known as Chavir (Azarbani et al., 2014) is a native plant in some parts of Iran (Sodeifian et al., 2011). The genus *Ferulago* belongs to the Apiaceae family and is used in folk medicine for sedative, tonic, digestive, and antiparasitic effects (Taran et al., 2010). There have been some published reports on its significant antibacterial, antioxidant, and antidiabetic properties, and, traditionally, it was added to dairy and oil ghee to prevent decay as a strong preservative and to increase the shelf life of dairy products besides adding a pleasant taste to them (Alizadeh et al., 2019; Azarbani et al., 2014).

This study aimed to investigate the effect of the ethanolic extract and essential oil of *F. angulata* on the protein, physicochemical, microbial, and sensory properties of probiotic yogurt and the viability of probiotic bacteria during storage time.

## 2 MATERIALS AND METHODS

### 2.1 Materials and bacterial strains

*Bacillus subtilis* (PTCC 1720), *Staphylococcus aureus* (PTCC 1112), and *Escherichia coli* (PTCC 1330) were collected from the Industrial Microorganism Collection Center (Iran). The yogurt starter culture (express 0/1 included *Streptococcus thermophilus* and *Lactobacillus delbrueckii* ssp. *bulgaricus*) and probiotic bacteria (*Lactobacillus acidophilus* (LA‐5) and *Bifidobacterium bifidum* (Bb‐12)) were obtained from Chr. Hansen, LTD, Denmark, and directly added to milk. All the microbial media and chemical materials were purchased from Merck, Germany.

### 2.2 Plant collection

The aerial parts of *F. angulata* subsp. were collected in July 2019 from Rayen Mountains, Kerman Province, Iran. The plant was identified and authenticated by the Agricultural Research and Promotion Center of Kerman.

### 2.3 Extraction procedure

To extract the essential oil (EO), the aerial parts *F. angulata* were air‐dried at ambient temperature in the shade; 150 g of them was distilled by a Clevenger‐type apparatus for 3 hr, FAEO was extracted and dried over anhydrous sodium sulfate, and stored at 4°C until analysis (Javidnia et al., 2006).

To extract the ethanolic extract (EE), the air‐dried parts of *F. angulata* were pulverized into the powdered form. The dried powder (30 g) was extracted by the maceration method with ethanol (EtOH), and separately at room temperature, and the solvents from the combined extracts were evaporated by the rotary system (Mottaghipisheh et al., 2014).

### 2.4 Analysis method

To analyze the components of FAEE and FAEO, a gas chromatograph (Agilent 7890A) coupled with mass spectrometry (Agilent 5975 C) GC/MS (HP‐5MS) equipped with a column (30 m in length, with an internal diameter of 0.25 mm and a thin layer thickness of 0.25 µm) was employed. The temperature profile was as follows: At first, the temperature of the oven was fixed on 45°C for 1 min and then increased to 300°C with a temperature rate of 5°C/min. The helium input flow rate was 1 ml/L (Sodeifian et al., 2011). The extract constituents were identified by the comparison of their retention indices relative to (C7‐C20) n‐alkanes and by comparison of their mass spectra with those of the internal reference mass spectra library (NIST and Wiley). The percentage of volatile compositions was calculated from the GC peak areas (Azarbani et al., 2019).

### 2.5 Disk diffusion antibacterial activity

The antibacterial activity of the samples was evaluated against *Bacillus subtilis*, *Staphylococcus aureus*, and *Escherichia coli* by the disk diffusion method (Azarbani et al., 2014). For this purpose, the agar diffusion method was used. The bacteria were cultured for 24 hr on the Mueller Hinton agar, and a suspension was prepared in 0.5 McFarland dilution (OD_625 nm_ = 0.1) in the Mueller Hinton broth. Then, 5 ml of each bacterial suspension was cultured with the spread plate method using a sterile swap, and blank disks containing 2,560 μg/ml of each EE/EO diluted with DMSO were placed on the culture medium. Subsequently, the inhibitory zone diameter was measured after 24 hr of incubation at 37°C. A tetracycline disk was used as the control disk (Moghtader et al., 2013).

### 2.6 Minimum inhibitory concentration (MIC) and minimum bactericidal concentration (MBC) test

The MIC and MBC test of FAEE and FAEO was determined by the tube dilution method described by Tabatabaei Yazdi et al. (2014) with some modifications. The bacterial suspensions were prepared the same as the disk diffusion test. Briefly, the bacterial dilation was prepared in nine sterile tubes. Eight tubes were used for serial dilution and one for control. All the bacteria were incubated at 37°C for 48 hr. After incubation, the tubes were examined for turbidity caused by the growth of inoculated microorganisms. All tubes with no growth were sampled and cultured to determine MBC. The tubes containing the lowest concentrations of FAEE and FAEO and in which no growth was observed in the relevant plate were considered as MBC (Tabatabaei Yazdi et al., 2014).

### 2.7 Yogurt preparation

Milk was mixed with skim milk powder (2%) and milk protein concentrate (0.5%) to standardize the fat (3.2%) and protein content to the desired level (10.5 w/w; Lee & Lucey, 2010). It was pasteurized at 90°C for 5 min and cooled to 43°C. Then, the yogurt starter (0.6 gr) and probiotic bacteria (0.4 gr) were added to milk (800 gr; EL Omari et al., 2020). To prepare the herbal probiotic yogurt, different concentrations of FAEE and FAEO were added to the milk (0.2% and 0.4% for FAEE, 0.01% and 0.03% for FAEO) in 100 g packages and then incubated at 43°C until reaching the pH value of 4.6. They were subsequently cooled down until 4°C (Sadeghi et al., 2017). The probiotic yogurt without FAEE and FAEO was selected as the control sample. After the production of probiotic yogurts, the samples were stored at the refrigerator temperature and analyzed during storage for 21 days (with four intervals; Ertem & Cakmakcı, 2018; Simon et al., 2018).

### 2.8 Protein tests

#### 2.8.1 Determination of total phenolic compounds (TPC)

The TPC was determined by Folin–Ciocalteu reagent. Briefly, 1 ml of the yogurt extract (a mixture of 10 gr of yogurt samples with 2.5 ml of distilled water) was mixed with 1 ml of 95% ethanol, 5 ml of distilled water, and 0.5 ml of the Folin–Ciocalteu reagent, and the contents of the tube were mixed thoroughly. Then, 1 ml of 1N 50% Na_2_CO_3_ was added, and the sample was incubated in the dark for 120 min at room temperature. Finally, the absorbance was measured at 725 nm with a spectrometer (T80UV/VIS Spectrometer, PG Instruments LTD; Ashrafi yourghanloo & Gheybi, 2019; Hassan et al., 2013). The results were expressed as μg of gallic acid equivalent (GAE) per g of sample (μg GAE/g of sample; Kim et al., 2019).

#### 2.8.2 FTIR test

Infrared (IR) or Fourier‐transform infrared (FTIR) spectroscopy has a large application range, from the analysis of small molecules or molecular complexes to the analysis of cells or tissues. It has also been increasingly applied to the study of proteins. This concerns the analysis of protein conformation, protein folding, and molecular details from protein active sites during enzyme reactions using reaction‐induced FTIR difference spectroscopy (Berthomieu & Hienerwadel, 2009). Herein, a Bruker Tensor 27 FTIR spectrometer equipped with a KBr beam splitter and DLaTGC detector was utilized. The samples were placed onto a silicon sample carrier and left to dry in ambient air for 30 min prior to data collection.

#### 2.8.3 Solubility of yogurt protein

The method used by Brückner‐Gühmann et al. (2019) with some modifications was adopted to analyze the solubility of yogurt protein samples. The yogurt samples were suspended at a concentration of 5% (w/w) in distilled water by magnetic stirring at room temperature for 1 hr. The pH was adjusted as required to pH 4 with 1N HCl. The suspension was centrifuged at 10,000 *g* for 10 min, and the protein content in the supernatant, as well as in the suspension before centrifugation, was determined according to the Kjeldahl method. The relation of the protein content in the suspension to the protein content in the suspension before centrifugation yields the protein solubility (Brückner‐Gühmann et al., 2019).

#### 2.8.4 Water absorption capacity

The water absorption capacity (WAC) of yogurt protein was determined using the protocol described by Rodríguez‐Ambriz, Martínez‐Ayala, Millán and Davila‐Ortiz (2005). The WAC (%) of the sample is calculated using by dividing the weight of the water absorbed by the weight of the protein sample (Al‐Shamsi et al., 2018).

### 2.9 Physicochemical tests

#### 2.9.1 Determination of the pH and titratable acidity of yogurts

The pH of yogurts was measured using a pH‐meter 766, and titratable acidity was determined by titration using 0.1 mol/L NaOH and phenolphthalein as an indicator, and is expressed as g of lactic acid per 100 g of yogurt (Marinaki et al., 2016).

#### 2.9.2 Syneresis measurement

Twenty‐five grams of unstirred yogurt was spread evenly on a Whatman No. 1 filter paper in a funnel. After 2 hr at 4°C, the volume of the serum isolated from yogurt in cc was recorded and expressed as the rate of syneresis (Ashrafi yourghanloo & Gheybi, 2019).

#### 2.9.3 Viscosity measurement

The viscosity of yogurt samples was determined at 4°C using spindle number 5 at a shear rate of 60 rpm (Ashrafi yourghanloo & Gheybi, 2019).

#### 2.9.4 Sensory evaluation

The sensory evaluation was conducted through consumer taste panels using a 5‐point hedonic scale (1 = least acceptable, 5 = extremely good). To perform this sensory evaluation, an eight‐person panel was used, in which the sensory evaluation of yogurt was performed using the general scoring method obtained by multiplying the scores given to the sensory indices in the relevant coefficients. The final evaluation indicator is the overall evaluation, and the maximum sum of sensory ratings is 50 (Sekhavatizadeh et al., 2015).

### 2.10 Microbial tests

#### 2.10.1 Survival of probiotic bacteria

The selective count of *B. bifidum* was performed using the TOS‐propionate agar medium supplemented with mupirocin lithium salt and sodium propionate. The plates were incubated anaerobically at 37°C for at least 72 hr. The MRS/CL/CIP Agar medium containing clindamycin and ciprofloxacin was utilized for the selective count of *L. acidophilus*. After incubation, viable numbers were enumerated using the surface culture technique (Sarvari et al., 2014).

#### 2.10.2 Mold and yeast test

To count the number of molds and yeasts, after 21 days of storage in the refrigerator, each probiotic yogurt sample was cultured in the YGC medium and the plates were aerobically incubated in a refrigerated incubator at 25°C for 3–5 days. After this period, the colonies were counted (El Omari et al., 2020).

### 2.11 Statistical analysis

The SPSS statistical software version 17 was used to analyze the data. One‐way analysis of variance (ANOVA) was performed to compare the means, and the Duncan test was used to examine the difference between the means at *p* < .05. The Excel software was used to plot the curves, and the hedonic five‐point method was adopted to analyze the sensory data.

## 3 RESULTS AND DISCUSSION

### 3.1 GC–MS analysis

The essential oil yields were 0.6% (v/w) based on the dry weights of the samples. The chemical composition of the FAEO is reported in Table 1, and 14 components were identified (72.42%). The major compounds of the oil were (Z)‐beta‐ocimene (14.22%), alpha‐pinene (12.61%), and germacrene B (10.52%).

**TABLE 1:** Components of *Ferulago angulata* essential oil

Components	RI	Percent	Components	RI	Percent
Alpha‐thujene	915	5.54	Thymol	1,280	2.66
Alpha‐pinene	935	12.61	Carvacrol	1,298	0.82
Myrcene	995	1.50	Longifolene	1,412	2.43
Limonene	1,024	2.10	Germacrene B	1,560	10.52
(Z)‐Beta‐ocimene	1,032	14.22	Spathulenol	1,578	3.31
Linalool	1,095	3.30	Diethyl Phthalate	1,590	7.51
Terpinen‐4‐ol	1,174	2.22	Hexadecanol	1,874	1.70

Abbreviation: RI, retention indices.

In the study by Rezazade et al. (2003), *F. angulata* volatile oil, which had been dried in the shade, was extracted by the water vapor distillation method and examined by a chromatographic gas device connected to a mass spectrometer detector, and its components were identified. In this work, 33 compounds that constituted 89.7% of the components were identified, of which 77.1% were monoterpenes and 12.6% were sesquiterpenes. The main identified compounds were alpha‐pinene, bornyl acetate, and cis‐ocimene, which were almost consistent with this study.

Azarbani et al. (2019) also investigated the phytochemicals, phenolic profiles, antioxidant, and antibacterial activities of *Ferulago macrocarpa* extracts from Lorestan (Iran) and observed that the volatile fraction of the leaves' extract comprised 48 constituents accounting for 94.91% of the total volatile amount. The main identified compounds were bornyl acetate (37.91%), o‐cymene (7.83%), 2‐hexanal (7.01%), camphene (5.57%), and α‐pinene (3.64%). The antibacterial activity of this plant may be attributed to the presence of terpinolene, thymol, and α‐pinene.

### 3.2 Inhibitory zone diameter

The results in Table 2 show that the inhibitory zone diameter of FAEO on *S. aureus*, *B. subtilis*, *L. bulgaricus*, and *S. thermophilus* bacteria was significantly greater than that of FAEE (*p* > .05). In *E. coli*, there was no significant difference in the antibacterial effect of the extract and the essential oil of *F. angulata* (*p* < .05). As for *B. subtilis*, the antibacterial effect of FAEO was equal to that of tetracycline (*p* < .05), and in other bacteria, the growth inhibition diameter in tetracycline was greater than that of FAEE and FAEO (*p* < .05). Most of the antimicrobial effect of FAEE was reported on *B. subtilis* (12 ± 0.50), FAEO on *S. aureus* (20 ± 0.80), and tetracycline, as the control, on *S. aureus* (24 ± 0.58).

**TABLE 2:** Results of inhibitory zone diameter test of growth of bacteria (millimeters) by the essential oil and extract of *Ferulago angulata*

Samples	*Staphylococcus aureus*	*Bacillus subtilis*	*Escherichia coli*	*Lactobacillus bulgaricus*	*Streptococcus thermophilus*
FAEE	9 ± 0.80 aB	12 ± 0.50 aC	9 ± 0.00 bB	7 ± 0.00 aA	7 ± 0.00 aA
FAEO	20 ± 0.80 bD	14 ± 0.50 bC	10 ± 1.00 bA	12.33 ± 0.58 bB	13 ± 1.00 bBC
Tetracycline	24 ± 0.58 cE	13.67 ± 0.58 bB	7 ± 0.45 aA	17.33 ± 0.58 cC	20.33 ± 0.58 cD

Note

The nonsimilar small letters indicate a significant difference in the column (*p* < .05). The large nonsimilar letters indicate a significant difference in the line (*p* < .05).

The results are shown as mean ± standard deviation.

Darderafshi et al. (2014) examined the effect of FAEO on the growth of *Staphylococcus aureus* during the production and maintenance of Iranian white cheese, and their results showed that the highest inhibitory effect was for 0.03% and 0.015% of essential oils. The results of the evaluation of pH changes during sample storage showed the effect of essential oil concentration on pH variation, such that this amount decreased with increasing the essential oil concentration.

Also in a study by Sadeghi, Akhondzadeh Basti, Noori, Khanjari and Partovi (2012) on green cumin essential oil, the best concentration of essential oil in terms of the effect of inhibiting the growth of staphylococcus and creating the desired taste in the product was 0.015%, which is consistent with the present study.

### 3.3 MIC and MBC analysis

The results of the MIC and MBC tests are given in Table 3, indicating that the FAEO had a greater effect on the bacteria and that a lower concentration was required to inhibit the growth of the bacteria. For MIC of FAEO, the concentrations of 160 μg/ml for *S. aureus* and *B. subtilis* and 640 μg/ml for *E. coli*, *L. bulgaricus*, and *S. thermophiles* were required; for MBC, the concentrations of 320 μg/ml for *S. aureus* and *B. subtilis*, and 1,280 μg/ml for *E. coli*, *L. bulgaricus*, and *S. thermophiles* were required. For MIC of the *F. angulate* extract, the concentration of 1,280 μg/ml, and for MBC, the concentration of 2,560 μg/ml were required.

**TABLE 3:** Results of MIC and MBC (μg/ml) microbial growth by extract and essential oil of *Ferulago angulate*

Bacteria	*Streptococcus thermophilus*		*Lactobacillus bulgaricus*		*Escherichia coli*		*Bacillus subtilis*		*Staphylococcus aureus*	
Samples	MIC	MBC	MIC	MBC	MIC	MBC	MIC	MBC	MIC	MBC
FAEE	1,280	2,650	1,280	2,650	1,280	2,650	1,280	2,650	1,280	2,650
FAEO	640	1,280	640	1,280	640	1,280	160	320	160	320

Abbreviations: MBC, minimum bactericidal concentration; MIC, minimum inhibitory concentration.

The results of the nongrowth zone diameter and MBC and MIC of FAEO and FAEE on pathogenic bacteria and yogurt starters showed that the antibacterial effect of essential oil is greater than the extract. Also, Gram‐positive bacteria were less resistant to extracts and essential oils. This is due to the structural difference in the wall of Gram‐positive bacteria compared to Gram‐negative ones; the Gram‐positive bacteria in their cell wall have a mucopeptide composition, while Gram‐negative bacteria have only a thin layer of mucopeptide, and most of the building wall in them is lipoprotein and lipopolysaccharide; therefore, Gram‐negative bacteria are more resistant. As a result, the higher resistance of Gram‐negative bacteria can be attributed to the presence of almost nonpermeable exogenous phospholipid membranes (Tabatabaei Yazdi et al., 2014).

### 3.4 Protein tests

#### 3.4.1 Analysis of TPC

**FIGURE 1:**
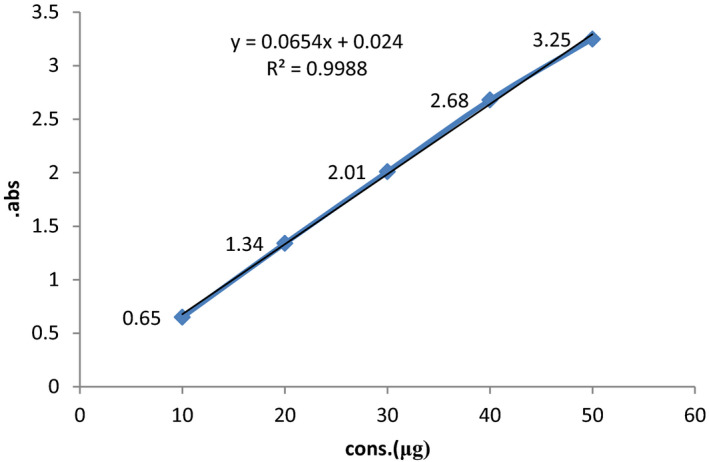
Gallic acid standard curve

Results in Table 4 showed that in all probiotic yogurts containing FAEE, the amount of phenolic compounds was lower than that of probiotic yogurts containing FAEO. In herbal probiotic yogurts, with increasing the extract concentration from 0.2 to 0.4, the amount of phenol increased (*p* < .05) and there was no significant difference in probiotic yogurt containing 0.01% and 0.03% of FAEO (*p* > .05). The lowest phenol content was measured in probiotic yogurts containing 0.2% of FAEE (45.10 ± 0.93 μg GAE/g). The highest amount of phenolic compounds was measured in probiotic yogurts containing 0.03% of FAEO (54.0 ± 0.65 μg GAE/g; *p* < .05). Figure 1 showed gallic acid standard curve.

**TABLE 4:** Comparison of TPC of herbal probiotic yogurts

Sample of probiotic yogurt	Phenol (μg GAE/g of sample)
With 0.2% of FAEE	45.10 ± 0.93 a
With 0.4% of FAEE	47.30 ± 0.70 b
With 0.01% of FAEO	53.40 ± 1.02 c
With 0.03% of EAEO	54.0 ± 0.65 c

Note

Nonsimilar small letters indicate a significant difference (*p* < .05).

The results are shown as mean ± standard deviation.

Esmaili et al. (2017) obtained the TPC of pomegranate essential oil and extract to be 53.14 and 37.68, respectively. Moreover, the high amount of phenolic compounds in the essential oil compared to the extract was attributed to the difference in the type of preparation and extraction method, their chemical nature, and the different effective compounds of each of them.

#### 3.4.2 FTIR test

**FIGURE 2:**
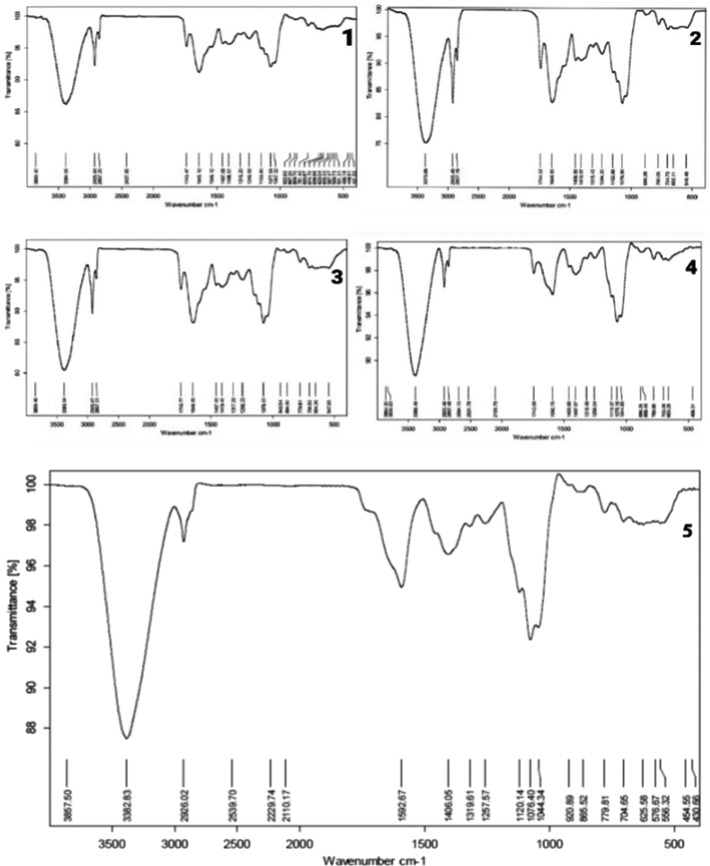
FTIR result (1) yogurt with 0/01% FAEO‐(2) yogurt with 0/03% FAEO‐(3) yogurt with 0/2% FAEE‐(4) yogurt with 0/4% FAEE‐(5) Control yogurt

According to the results of Hosseini Shirazi et al. (2005), based on Figure 2, the widest peak belongs to the N‐H stretching groups, and the peak in the 1,500 range belongs to the C = O and C‐N stretching groups and the N‐H bending group. The range of 1,000 belongs to the C = O, C–N, and N–H bending groups. By comparing the control yogurt with other herbal yogurts, one can conclude that the use of this amount of essential oil and extract does not change the structure of proteins.

#### 3.4.3 Analysis of the solubility and WAC of yogurt protein

Table 5 presents the results of the solubility and WAC of yogurt protein. There is a significant difference in the protein solubility and the percentage of WAC of different treatments of probiotic yogurts containing the extract and essential oil of *F. angulata* (*p* < .01). The solubility of the protein in probiotic yogurts containing FAEO was significantly higher than that of probiotic yogurts containing the extract (*p* < .05). Compared to the control treatment, adding FAEE (0.2% and 0.4%) to probiotic yogurts significantly reduced protein solubility, while in probiotic yogurts containing FAEO (0.01% and 0.03%), it significantly increased protein solubility compared to the control treatment (*p* < .05). The lowest and highest protein solubility belonged to probiotic yogurts containing 0.2% FAEE (0.34 ± 0.04) and those containing 0.03% FAEO (0.58 ± 0.03).

**TABLE 5:** Analysis of solubility and WAC of protein yogurt

Sample of probiotic yogurt	WAC (%)	Protein solubility
Control	2.27 ± 0.10 a	0.47 ± 0.03 b
With 0.2% of FAEE	4.37 ± 0.53 bc	0.34 ± 0.04 a
With 0.4% of FAEE	4.66 ± 0.80 c	0.37 ± 0.02 a
With 0.01% of FAEO	3.35 ± 0.60 b	0.50 ± 0.04 b
With 0.03% of EAEO	3.72 ± 0.42 bc	0.58 ± 0.03 c

Note

Nonsimilar small letters indicate a significant difference (*p* < .05).

The results are shown as mean ± standard deviation.

Brückner‐Gühmann et al. (2019) in their research on enrichment of yoghurt with oat protein fractions: Structure formation, textural properties, and sensory evaluation explained generally, and three factors affect the compatibility of proteins: (a) different solubility in the solvent, (b) different molecular weight, and (c) differences in the conformation. Protein solubility of both oat protein concentrate (OPC) and oat protein isolate (OPI) is pH dependent. At pH 7, which is close to the pH of milk, solubility of OPC and OPI was found to be around 30%.

In all probiotic yogurts containing FAEE and FAEO, the WAC of the protein increased compared to the control treatment (*p* < .05). The addition of FAEE compared to FAEO significantly increased the WAC of protein in probiotic yogurts (*p* < .05). The highest protein WAC was measured in probiotic yogurts containing 0.4% of the extract (4.66 ± 0.80).

WAC is related to the ability of a protein to hold water in the yogurt gel structure and according to results of Kim et al. (2019) who worked on effects of lotus (*Nelumbo nucifera*) leaf on quality and antioxidant activity of yogurt during refrigerated storage, and WAC of LL yogurts was higher than that of the control during storage (*p* < .05).

### 3.5 Physicochemical tests

#### 3.5.1 pH, titratable acidity, viscosity, and syneresis of yogurts

According to Table 6, on the first and the 21st days, the pH of different herbal probiotic yogurt treatments did not differ significantly (*p* < .05). The results show that the pH changes in herbal probiotic yogurts were significant in the control treatment over time (*t* = 3.062 and *p* = .038). Over time, the pH in the control treatment ranging from 4.25 ± 0.10 to 4.00 ± 0.10 decreased. In other treatments, pH changes were not significant over time.

**TABLE 6:** The results of pH, titratable acidity, viscosity, and syneresis of herbal probiotic yogurts

Sample of probiotic yogurts	pH		Acidity (Dornic degree)		Viscosity (Pa.s)		Syneresis (cc)	
	Day 1	Day 21	Day 1	Day 21	Day 1	Day 21	Day 1	Day 21
Control	4.25 ± 0.10 aB	4.00 ± 0.10 aA	120 ± 1 aB	126 ± 2 aA	0.37 ± 0.05 aB	0.18 ± 0.03 aA	1.55 ± 0.07 cA	4.25 ± 0.07 hB
With 0.2% of FAEE	4.12 ± 0.08 aA	4.02 ± 0.08 aA	131 ± 3 cA	132 ± 1 cA	0.41 ± 0.03 abB	0.23 ± 0.05 aA	0.32 ± 0.05 aA	3.35 ± 0.06 dB
With 0.4% of FAEE	4.14 ± 0.09 aA	4.01 ± 0.06 aA	130 ± 1 cA	132 ± 1 cA	0.40 ± 0.06 abB	0.21 ± 0.02 aA	0.40 ± 0.03 bA	3.10 ± 0.13 cB
With 0.01% of FAEO	4.14 ± 0.07 aA	4.03 ± 0.04 aA	129 ± 1 cA	129 ± 1 bA	0.51 ± 0.02 cB	0.37 ± 0.07 bA	0A	2.10 ± 0.11 aB
With 0.03% of EAEO	4.22 ± 0.12 aA	4.07 ± 0.11 aA	125 ± 2 bA	128 ± 2 abA	0.49 ± 0.07 bcB	0.36 ± 0.04 bA	0A	2.20 ± 0.06 aB

Note

The nonsimilar small letters indicate a significant difference in the column (*p* < .05). The large nonsimilar letters indicate a significant difference in the line (*p* < .05).

The results are shown as mean ± standard deviation.

The control treatment had the highest acidity on the first day (120 ± 1D) and on the 21st day (126 ± 2D; *p* < .05). The addition of FAEE was more effective in increasing the acidity of probiotic yogurts than FAEO (*p* < .05). The addition of FAEO from 0.01% to 0.03% caused a decrease in acidity (*p* < .05), but there was no significant difference in the acidity of probiotic yogurts containing 0.2% and 0.4% of the extract (*p* < .05). The highest acidity was measured on the first and 21st days in probiotic yogurts containing 0.4% FAEE (Table 6).

In all probiotic yogurts containing essential oils and extracts of the *F. angulata* plant, the viscosity increased compared to the control treatment. The addition of FAEO was more effective in increasing viscosity compared to FAEE (*p* < .05). There was no significant difference in the viscosity of probiotic yogurts containing 0.2% and 0.4% *F. angulata* (*p* < .05). The treatment containing 0.01% of essential oil had the highest viscosity on the first and 21st days (0.51 ± 0.02 pa.s & 0.37 ± 0.07 pa.s). Over time, viscosity was significantly reduced in all probiotic yogurts containing the extracts and essential oils of *F. angulata* (*p* < .05; Table 6).

The rate of syneresis in treatments containing 0.01 and 0.03% FAEO was zero, while the control treatment had the highest (1.55 ± 0.07 cc) syneresis rate. Over time, the syneresis rate was significantly increased in all probiotic yogurts containing extracts and essential oils of *F. angulata* (*p* < .05; Table 6).

Adding plant extracts to probiotic yogurt leads to a significant increase in acidity compared to the control sample, which is because fermenting yogurt with plant extracts increases the metabolic activity of yogurt bacteria and acidity due to the production of organic acids of lactic acid bacteria. In addition, over time, the acidity of all treatments increases significantly, which is due to the increase in storage time and the continuation of the process of lactose fermentation by the starter and probiotic bacteria, increasing acidity due to the accumulation of acids such as lactic acid and formic acid (Ghalemousiani et al., 2017). In this study, the relationship between pH and acidity in different treatments of probiotic yogurt was reversed. In other words, by increasing pH, acidity decreased.

In this study, the viscosity of the product increased with the addition of essential oil and extract. Similarly, Kim et al. (2019) concluded that viscosity improved when LL (lotus leaf) powder was added to the yogurts (*p* < .05). An at least 4‐fold higher viscosity was observed in LL yogurts than in the control during storage. Polyphenols can bind to proteins and form protein–polyphenol complexes. The abundant phenolic compounds in LL interacted with milk proteins such as casein in the yogurt matrix, resulting in higher viscosity than that in the control.

According to Ashrafi yourghanloo and Gheybi (2019), increasing the denaturation of whey proteins improves water retention capacity and thus reduces dehydration. The tendency to hydrate is a function of the concentration of whey proteins. By increasing the ratio of whey proteins along with the reduction in casein micelles, the main factor in gel formation, dehydration (syneresis) increases. They also reported that by adding 5% and 10% of dill extract to yogurt, the amount of syneresis decreases and increases, respectively, compared to the control sample.

### 3.6 Analysis of sensory evaluation

**FIGURE 3:**
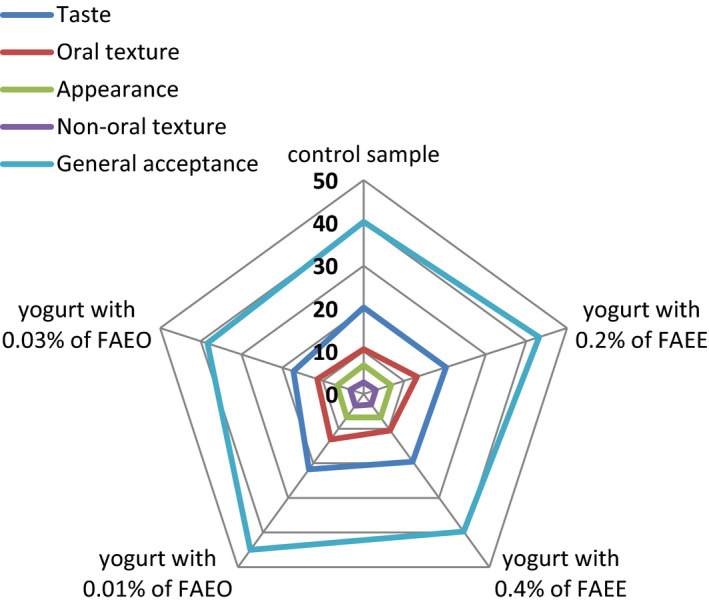
Comparison of sensory properties of herbal probiotic yogurts

According to the results, the oral texture of probiotic yogurts containing 0.4% of extract and 0.03% of FAEO was not significantly different from the control treatment (*p* < .05). In these treatments, the oral texture score was higher than probiotic yogurts containing 0.2% extract and 0.01% FAEO (*p* < .05). In evaluating the sensory characteristics of the nonoral tissue, after the control treatment, the highest score was obtained by probiotic yogurts containing 0.01% and 0.03% essential oil of *F. angulata*. In terms of the general acceptance of the evaluators, the most desirable treatment was the probiotic yogurt containing 0.03% of FAEO. Comparison of sensory properties of herbal probiotic yogurts has been shown in Figure 3.

**TABLE 7:** Comparison of the survival of *Lactobacillus acidophilus* (CFU/g) in herbal probiotic yogurts

Sample of probiotic yogurts	Day 1	Day 7	Day 14	Day 21
Control	1.92 × 108 ± 1.5 × 106 aC	4.54 × 106 ± 8.0 × 103 aB	8.10 × 105 ± 9.0 × 103 aA	1.14 × 105 ± 1.0 × 103 aA
With 0.2% of FAEE	2.55 × 108 ± 1.7 × 106 cC	2.00 × 107 ± 8.0 × 105 dB	1.82 × 106 ± 7.0 × 104 bA	8.49 × 105 ± 1.0 × 103 cA
With 0.4% of FAEE	2.34 × 108 ± 2.0 × 106 bD	8.18 × 106 ± 9.0 × 103 bC	2.81 × 106 ± 1.0 × 105 cB	8.82 × 105 ± 2.0 × 103 dA
With 0.01% of FAEO	3.20 × 108 ± 1.3 × 106 eD	3.00 × 107 ± 1.0 × 106 eC	3.12 × 106 ± 1.0 × 105 eB	8.17 × 105 ± 3.0 × 103 bA
With 0.03% of EAEO	3.01 × 108 ± 6.0 × 106 dD	1.36 × 107 ± 1.5 × 105 cC	3.50 × 106 ± 1.0 × 105 eB	9.10 × 105 ± 8.0 × 103 eA

Note

The nonsimilar small letters indicate a significant difference in the column (*p* < .05). The large nonsimilar letters indicate a significant difference in the line (*p* < .05).

The results are shown as mean ± standard deviation.

Azizkhani and Parsaeimehr (2018) worked on probiotic survival, antioxidant activity, and sensory properties of yogurt flavored with herbal (peppermint, basil, and zataria) essential oils and concluded that peppermint and basil samples showed both good antiradical activity and sensory acceptability. In the sensory tests, yogurt samples were evaluated for appearance, flavor, texture, and overall acceptability. The mean scores for the appearance of basil and peppermint treated yogurt were higher than the control yogurt. The mean scores for the appearance of probiotic yogurt with basil and peppermint were within the acceptable range, but there was no significant difference between the types of yogurt (*p* > .05). Also, the mean scores for zataria yogurt were significantly lower than the control (*p* < .05).

### 3.7 Microbial tests

#### 3.7.1 Survival of probiotic bacteria

The microbiological analysis of the yogurt samples in Tables 7, 8and 8 demonstrates the viability of the probiotic culture during storage. The survival of *L. acidophilus* in the probiotic yogurt containing the extract and essential oil of the *F. angulata* plant was significantly higher than in the control treatment (*p* < .05). The highest survival rate of *L. acidophilus* was measured on the first and 7th days in probiotic yogurts containing 0.1%, and on the 14th and 21st days in probiotic yogurts containing 0.03%, respectively. In general, *L. acidophilus* decreased in all treatments over time. In the treatment of the plant probiotic control and yogurt containing 0.2 FAEE, the percentage of survival changes on the 14th and 21st days was not significant (Table 7, 8).

**TABLE 8:** Comparison of the survival of *Bifidobacterium bifidum* (CFU/g) in herbal probiotic yogurts

Sample of probiotic yogurts	Day 1	Day 7	Day 14	Day 21
Control	2.08 × 10^8^ ± 1.0 × 10^6^ aC	6.00 × 10^7^ ± 1.0 × 10^6^ aB	1.21 × 10^7^ ± 9.0 × 10^4^ aA	6.93 × 10^5^ ± 1.5 × 10^3^ aA
With 0.2% of FAEE	2.67 × 10^8^ ± 1.2 × 10^6^ cC	7.30 × 10^7^ ± 9.0 × 10^5^ dB	2.31 × 10^7^ ± 1.0 × 10^5^ bA	1.14 × 10^6^ ± 1.10 × 10^5^ cA
With 0.4% of FAEE	3.19 × 10^8^ ± 5.0 × 10^6^ bD	7.90 × 10^7^ ± 8.0 × 10^5^ bC	3.00 × 10^7^ ± 1.0 × 10^6^ cB	2.01 × 10^6^ ± 1.0 × 10^4^ dA
With 0.01% of FAEO	4.12 × 10^8^ ± 1.0 × 10^6^ eD	8.10 × 10^7^ ± 6.0 × 10^5^ eC	2.52 × 10^7^ ± 6.0 × 10^4^ eB	2.52 × 10^6^ ± 1.70 × 10^4^ bA
With 0.03% of EAEO	4.16 × 10^8^ ± 2.0 × 10^6^ dD	8.30 × 10^7^ ± 1.6 × 10^6^ cC	2.83 × 10^7^ ± 1.0 × 10^5^ eB	3.07 × 10^6^ ± 1.00 × 10^4^ eA

Note

The nonsimilar small letters indicate a significant difference in the column (*p* < .05). The large nonsimilar letters indicate a significant difference in the line (*p* < .05).

The results are shown as the mean ± standard deviation.

**TABLE 9:** Results of mold and yeast counting of herbal probiotic yogurts

Sample of probiotic yogurt	Mold and yeast count (CFU/g)
Control	1.9 × 10^3^ ± 5 × 10 d
With 0.2% of FAEE	5.2 × 10^2^ ± 4 × 10 b
With 0.4% of FAEE	1.8 × 10^2^ ± 3 × 10 a
With 0.01% of FAEO	1.2 × 10^3^ ± 8 × 10 c
With 0.03% of EAEO	5.7 × 10^2^ ± 7 × 10 b

Note

Nonsimilar small letters indicate a significant difference (*p* < .05).

The results are shown as mean ± standard deviation.

The survival of *B. bifidum* in probiotic yogurts containing the extracts and essential oils of the *F. angulata* plant was significantly higher than the control treatment (*p* < .05). On the first, 7th, and 21st days, the highest survival of *B. bifidum* was measured in probiotic yogurts containing 0.03% of FAEO and on the 14th day in probiotic yogurts containing 0.4% of FAEE. In general, in all treatments, the survival time of the bacterium *B. bifidum* decreased, and the lowest life expectancy was measured on the 21st day and the highest life expectancy on the first day (Table 8).

According to the tables, it can be concluded that the best time to consume yogurts is until the 14th day, because the number of probiotic bacteria after that falls below 10^6^ CFU/ml.

Marhamatizadeh et al. (2013) investigated the effect of olive leaf extract on the growth and survival of *L. acidophilus* and *B. bifidum* in milk and probiotic yogurt over 21 days in the refrigerator and achieved similar results. In this study, olive leaf extract at the concentrations of 2%, 4%, and 6% was added to the samples. The results showed that the number of *L. acidophilus* and *B. bifidum* in samples containing olive leaf extract is significantly higher than the control sample. There was also a positive relationship between bacterial growth and increased concentration of the olive leaf extract.

Also, Ghalemousiani et al. (2017) found that the addition of plant extracts to probiotic yogurt containing *Lactobacillus paracasei* significantly increases the survival of probiotic bacteria compared to the control yogurt (probiotic yogurt without extract) due to the phenolic compounds in plant extracts. These have been shown to play a stimulating role in improving the growth of yogurt starter and probiotic bacteria. It was also noted that as the amount of dissolved oxygen in the product environment decreases, the bioavailability of probiotics increases. Moreover, phenolic compounds improve the growth of probiotic bacteria in the absence of oxygen.

Research also shows that, in general, the rate of loss of *B. bifidum* is higher than that of *L. acidophilus* and other lactic acid probiotics, and its growth and proliferation rate are lower in the product. This can be attributed to the higher sensitivity of these bacteria to oxygen, high acidity, and low pH (Marhamatizadeh et al., 2010).

#### 3.7.2 Analysis of mold and yeast count

According to Table 9, the highest number of mold and yeast counts (1.9 × 10^3^±5 × 10 CFU/g) was measured in the control treatment. With increasing the concentrations of extracts and essential oils, the mold and yeast count of probiotic yogurts decreased. The lowest (1.8 × 10^2^±3 × 10 CFU/g) mold and yeast counts were measured in probiotic yogurts containing 0.4% FAEE.

## 4 CONCLUSION

The results demonstrated that there is a significant difference in the amount of TPC, protein solubility, and WPC of different probiotic yogurt treatments containing the extract and essential oil of *F. angulata* (*p* < .01). There was also a significant difference in the acidity, viscosity, and syneresis of samples containing the extract and essential oil of *F. angulata* (*p* < .01, *p* > .05, and *p* > .01, respectively). Therefore, the addition of FAEE and FAEO significantly increased the TPC, acidity, viscosity, and protein solubility of the samples. pH changes in different treatments over time were not significant (*p* < .05).

In microbial tests, the results indicated a significant increase in the diameter, inhibition of the growth of *S. aureus*, *B. subtilis*, *E. coli*, *L. bulgaricus*, and *S. thermophiles*, and the survival of *L. acidophilus* and *B. bifidum* by adding the essential oil and extract of *F. angulate*. Adding FAEE and FAEO to yogurt also reduced the counted mold and yeast due to their antimicrobial composition (*p* < .01). Generally, according to the results of protein, physicochemical, microbial, and sensory tests of plant probiotic yogurts, it can be concluded that adding 0.03% essential oil is the best treatment and according to the amount of probiotic bacteria, the best time to consume it is until the 14th day.

## CONFLICT OF INTEREST

The authors declare that there is no conflict of interest regarding the publication of this paper.
